# Crystal structures of two 2,3-di­ethyl­naphtho­[2,3-*g*]quinoxaline-6,11-dione derivatives

**DOI:** 10.1107/S2056989017009641

**Published:** 2017-07-07

**Authors:** Craig M. Forsyth, Craig L. Francis

**Affiliations:** a School of Chemistry, Monash University, Clayton Victoria 3800, Australia; b Biomedical Synthetic Chemistry Group, CSIRO, Clayton, Victoria 3169, Australia

**Keywords:** crystal structure, naphtho­quinoxaline-6,11-dione, hydrogen bonding, π–π inter­action

## Abstract

Two new 5,12-disubstituted 2,3-di­ethyl­naphtho­[2,3-*g*]quinoxaline-6,11-dione compounds were readily synthesized from the commercial dye quinizarin. For 2,3-diethyl-5,12-di­hydroxy­naphtho­[2,3-*g*]quinoxaline-6,11-dione, (II), C_20_H_16_N_2_O_4_, the mol­ecule displays a near planar conformation and both hy­droxy groups participate in intra­molecular O—H⋯O(carbon­yl) hydrogen bonds. In the crystal, π–π ring inter­actions [minimum ring centroid separation = 3.5493 (9) Å] form stacks of co-planar mol­ecules down the *c* axis, while only minor inter­molecular C—H⋯O inter­actions are present. In contrast, in 2,3-diethyl-5,12-bis­(piperidin-1-yl)naphtho­[2,3-*g*]quinoxaline-6,11-dione, (IV), C_30_H_34_N_4_O_2_, which contains two independent, but similar, mol­ecules in the asymmetric unit, the polycyclic cores have a significant twist, with dihedral angles of 29.79 (6) and 29.31 (7)° between the terminal rings and only minor inter­molecular C—H⋯O hydrogen-bonding inter­actions are present. Electron density associated with additional solvent mol­ecules disordered about a fourfold axis was accounted for using the SQUEEZE procedure in *PLATON* [Spek (2015 ▸). *Acta Cryst.* C**71**, 9–18].

## Abstract 2

The syntheses and crystal structures of two 2,3-di­ethyl­naphtho­quinoxaline-6-11-dione derivatives are described. Mol­ecules of C_20_H_16_N_2_O_4_ (II) are near planar and form stacks down the *c* axis through π–π ring inter­actions. In the substituted derivative, C_30_H_34_N_4_O_2_ (IV), the polycyclic cores have a significant twist and only minor inter­molecular C—H⋯O hydrogen-bonding inter­actions are present.

## Chemical context   

**Figure 1:**
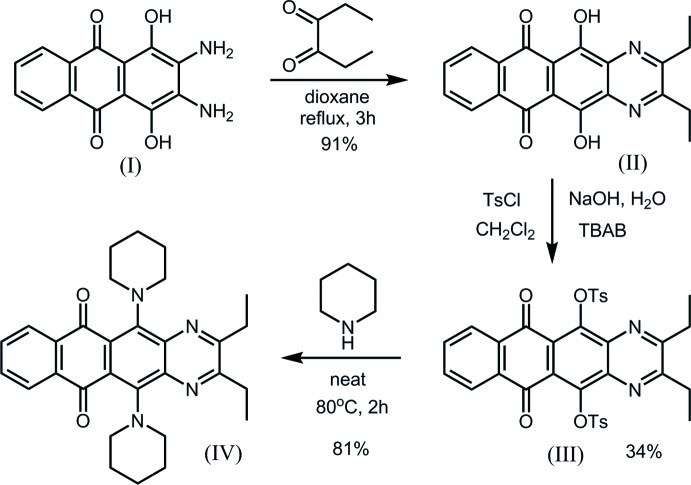
Reaction scheme for the synthesis of compound (IV) *via* inter­mediate compound (II).

As part of a program aimed at the identification of new heterocyclic compounds for organic electronic applications, we sought new or uncommon ring systems that could be synthesized conveniently from cheap, readily available starting materials. In this context, we noted that 2,3-di­amino-1,4-di­hydroxy­anthracene-9,10-dione (I) had been prepared from the inexpensive dye quinizarin (1,4-di­hydroxy­anthra­quinone) (Shchekotikhin *et al.*, 2005 ▸). The di­amine (I) appeared to us to be a convenient synthetic building block for fusion of di­aza-heterocycles onto the anthra­quinone core. Our reaction of the di­amine (I) with hexane-3,4-dione in dioxane afforded the 2,3-diethyl-5,12-di­hydroxy­naphtho­[2,3-*g*]quinoxaline-6,11-dione (II). In exploring the chemistry of compound (II), we found that conversion of the hy­droxy groups to the corresponding tosyl­ates gave (III) and subsequent reaction with an excess of piperidine afforded 2,3-diethyl-5,12-bis­(piperidin-1-yl)naphtho­[2,3-*g*]quinoxaline-6,11-dione (IV). The reaction scheme for the total synthesis is shown in Fig. 1 ▸ and the crystal structures of both the inter­mediate compound (II) and compound (IV) are reported herein.
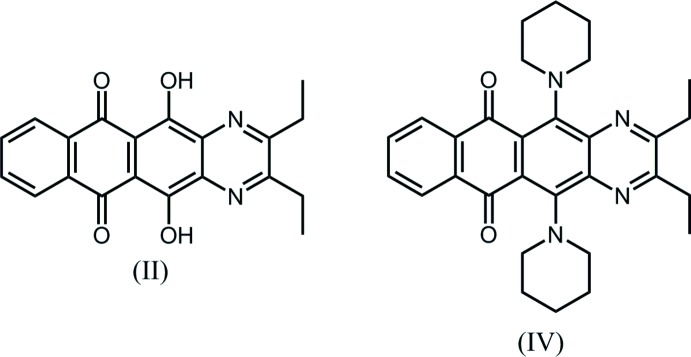


## Structural commentary   

**Figure 2:**
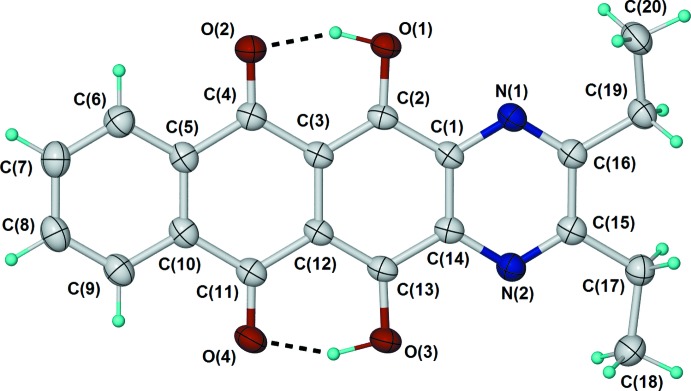
Mol­ecular conformation and atom-numbering scheme for (II), with displacement ellipsoids shown at the 50% probability level. Intra­molecular hydrogen bonds shown as dashed lines.

The mol­ecular structure of compound (II) is shown in Fig. 2 ▸. The naphtho­quinoxaline core is essentially planar [maximum deviation 0.0739 (11) Å for N1], with a dihedral angle of 4.60 (8)° between the terminal rings of the mol­ecule. Present in the mol­ecule are two intra­molecular O—H⋯O hydrogen-bonded ring systems formed by the hy­droxy and carbonyl substituents (Table 1 ▸). The two ethyl groups are approximately coplanar with the polycyclic core [torsion angles: N1—C16—C19—C20, 14.3 (2)° and N2—C15—C17—C18, −1.9 (2)°].

**Figure 3:**
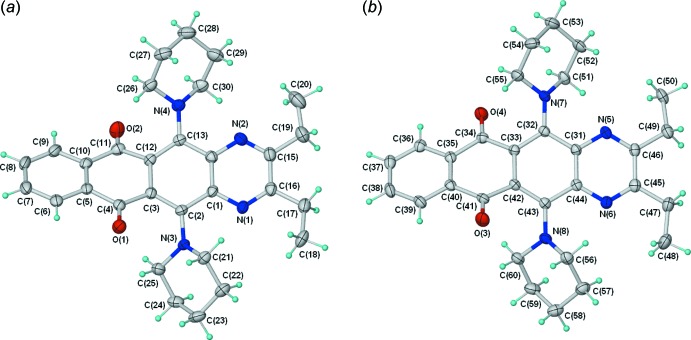
Mol­ecular conformation and atom-numbering scheme for the two independent mol­ecules [(*a*) mol­ecule 1 and (*b*) mol­ecule 2] in the asymmetric unit of (IV), with displacement ellipsoids shown at the 50% probability level.

The mol­ecular structure of compound (IV) contains two independent, but conformationally very similar mol­ecules (mol­ecule 1 and mol­ecule 2) (Fig. 3 ▸). In contrast to (II), the naphtho­quinoxaline core of (IV) is significantly twisted, as shown by the dihedral angles between the mean planes of the two terminal six-membered rings [29.79 (6) and 29.31 (7)°]. There is a corresponding twisting of the two central six-membered rings, presumably resulting from repulsion between neighbouring piperidin-1-yl and carbonyl moieties. The C—N bonds form angles of between 32.3 and 44.5° relative to the neighbouring C=O bonds.

## Supra­molecular features   

**Figure 4:**
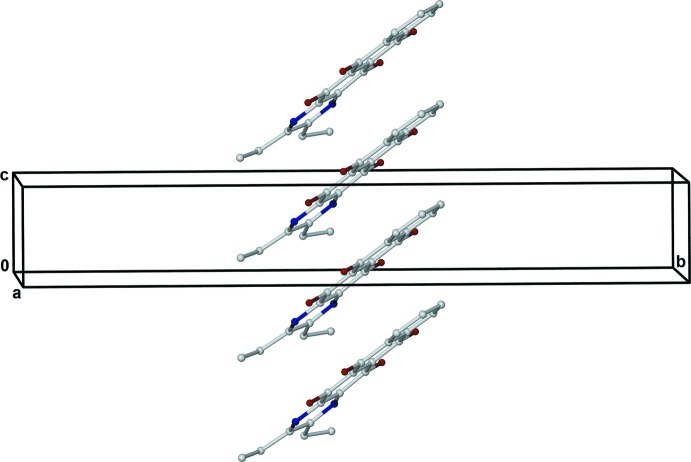
A view of an off-set vertical stack of mol­ecules of (II), extending along *c*.

**Figure 5:**
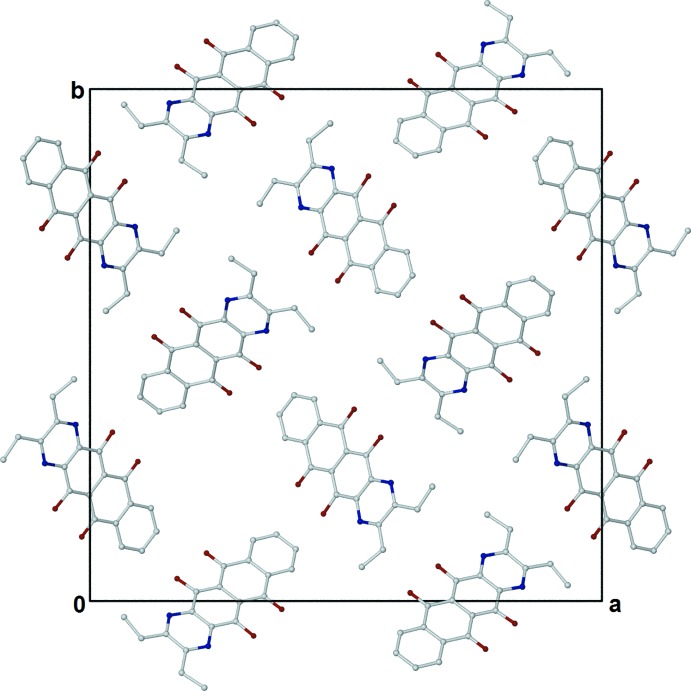
The packing in the unit cell of (II) as viewed along the *c* axis, with C-bound H atoms omitted.

In the crystal, mol­ecules of (II) form canted head-to-head π–π associated mol­ecules with ring centroid separations of 3.5493 (9) Å (*Cg*1⋯*Cg*2^iii^) [symmetry code: (iii): −*x*, −*y*, −*z* − 1], and 3.6064 (10) Å for (*Cg*2⋯*Cg*3^iv^) [symmetry code (iv): −*x*, −*y*, −*z* + 1] where *Cg*1, *Cg*2 and *Cg*3 are the centroids of the six-membered rings defined by atoms N1/N2/C1/C14–C16, C1–C3/C12–C14 and C3–C5/C10–C12, respectively. These slight variations in π–π separations result from the mol­ecules being off-set by one six-membered ring along the long mol­ecular axis and by approximately half a six-membered ring along the short mol­ecular axis. The result is the formation of stacks along the *c* axis with an inter-planar separation of *ca* 3.41 Å (Fig. 4 ▸). The packing viewed down the *c* axis is shown in Fig. 5 ▸ and displays an approximately orthogonal arrangement of the mol­ecular stacks. Present also in the crystal structure are two minor inter­molecular C—H⋯O inter­actions linking the stacks (aromatic C8—H⋯O1^i^ and methyl­ene C19—H⋯O4^ii^; Table 1 ▸).

**Figure 6:**
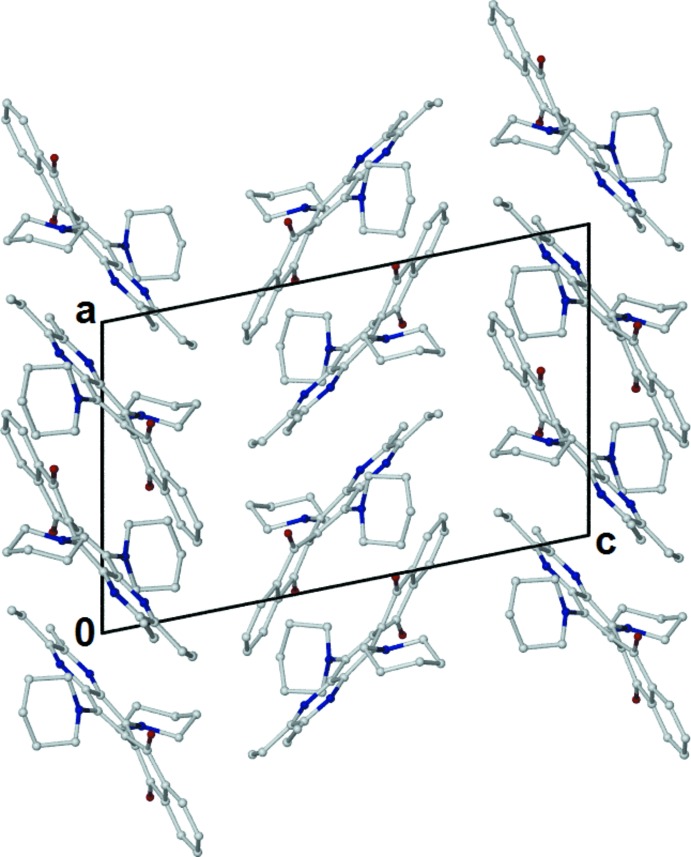
The packing in the unit cell of (IV) as viewed along the *b* axis, with H atoms omitted.

In contrast, the crystal packing of (IV) (Fig. 6 ▸) involves no π–π ring inter­actions [minimum *Cg⋯*Cg** separation = 3.9440 (9) Å between inversion-related mol­ecules]. There is only one significant inter­molecular hydrogen-bonding inter­action involving only mol­ecule 2: piperidin-1-yl C56—H⋯O3^i^ = 3.1765 (19) Å [symmetry code (i) −*x*, −*y* + 1, −*z* + 1], giving inversion-related dimers (Table 2 ▸).

## Database survey   

A search of the Cambridge Structural Database (V5.38; Groom *et al.*, 2016 ▸) for the naphtho­quinoxaline core gave three matches each having an additional fused six-membered ring, including the unsubstituted *N*-hetero­penta­cene pyrazino­[2′,3′;6,7]naphtho­[2,3-*g*]quinoxaline-6,13-dione (ref code AROCAM; Liang *et al.*, 2010 ▸) and two 13-chloro-6-methyl­carboxyl­ato-naphtho­[2,3-*b*]phenazine-7,12-diones (ref codes ABUVAW and ABUVEA; Chou *et al.*, 2011 ▸). Each of these examples have planar, or only slightly twisted (*ca* 12°) polycyclic cores and adopt off-set π–π stacked supra­molecular structures.

## Synthesis and crystallization   

**(i) 2,3-Diethyl-5,12-di­hydroxy­naphtho­[2,3-g]quinoxaline-6,11-dione, (II)**

Compound (II) was prepared using the procedure of Shchekotikhin *et al.* (2005 ▸), as follows. To a stirred mixture of di­amine (I) (1.35 g, 5 mmol), hexane-3,4-dione (3.0 ml, 2.85g, 25mmol), and 1,4-dioxane (30 ml) was heated at reflux for 3 h. The mixture was cooled and the resulting crystalline solid was collected by filtration and washed with diethyl ether to afford the title compound (1.58g, 91% yield) as rust-red needles, m.p. 507–509 K (found: *M* ^+^ 348.1102; C_20_H_16_N_2_O_4_ requires *M* ^+^ 348.1105). ^1^H NMR (CDCl_3_, 500 MHz) δ 8.42 (2H, *m*, ArH), 7.85 (2H, *m*, ArH), 3.18 (4H, *q*, *J* = 7.5Hz, CH_2_), 1.47 (6H, *t*, *J =* 7.5 Hz, CH_3_); ^13^C NMR (CDCl_3_, 125 MHz) δ 184.11, 161.55, 159.86, 139.09, 134.56, 133.80, 127.28, 109.12, 28.94, 12.84. Red–orange needles of (II). Crystals suitable for X-ray structure determination were grown from an acetone solution.

**(ii) 2,3-Diethyl-6,11-dioxo-6,11-di­hydro­naphtho­[2,3-g]quinoxaline-5,12-diyl bis­(4-methyl­benzene­sulfonate) (III)**

Compound (III) was prepared using the procedure of Zielske (1987 ▸). A mixture of diol (II) (1.04g, 3.0 mmol), *p*-toluene­sulfonyl­chloride (2.92 g, 15.3 mmol), CH_2_Cl_2_ (100 ml), aqueous sodium hydroxide (0.5%, 208 mL, 25.3 mmol), and tetra­butyl­ammonium bromide (4.96 g, 15.3 mmol) was stirred rapidly for 24 h at room temperature. The organic phase was set aside and the aqueous phase was extracted with di­chloro­methane (50 ml). The combined organic phase was washed with water (3 × 200 ml), saturated brine (50 ml), and dried over MgSO_4_. After filtration, the solvent was removed by evaporation under reduced pressure. The residual red–brown gum (3.63 g) was purified by chromatography over silica gel. Elution with 0–10% ethyl acetate in di­chloro­methane and evaporation afforded compound (III) (661 mg, 34%) as a honeycomb-coloured powder (found: *M* ^+^ 656.1278; C_34_H_28_N_2_O_8_^32^S_2_ requires *M* ^+^ 656.1282.) ^1^H NMR (CDCl_3_, 400 MHz) δ 8.05 (2H, *m*, ArH), 7.82 (4H, *d*, *J* = 8Hz, ArH), 7.75 (2H, *m*, ArH), 7.30 (4H, *d*, *J* = 8Hz, ArH), 2.84 (4H, *q*, *J* = 7.4 Hz, 2 × CH_2_), 2.45(6H, *s*, 2 × ArCH_3_), 1.25 (6H, *t*, *J* = 7.4 Hz, 2 × CH_3_); ^13^C NMR (CDCl_3_, 50 MHz) δ 180.81, 161.20, 145.03, 138.66, 134.43, 134.32, 134.03, 129.59, 128.73, 127.02, 125.78, 28.34, 21.69, 11.15.

**(iii) 2,3-Diethyl-5,12-bis­(piperidin-1-yl)naphtho­[2,3-** ***g*** **]quinoxaline- 6,11-dione, (IV)**

**Figure 7:**
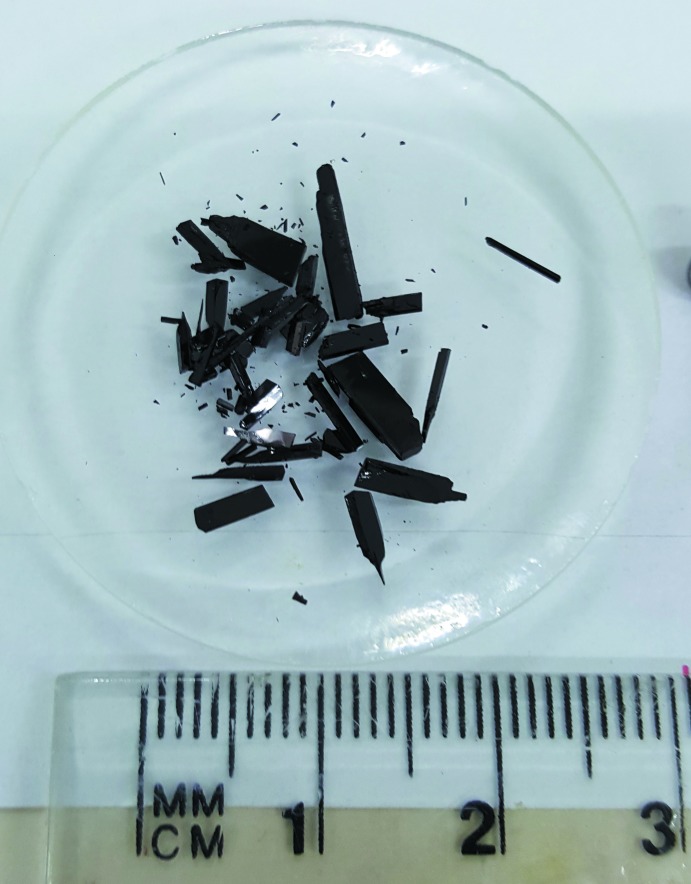
A photograph of crystals of (IV)

Compound (IV) was prepared by modifying the procedures of Zielske (1987 ▸) and Melliou *et al.* (2001 ▸). A stirred mixture of the bis-tosyl­ate (III) (550 mg, 0.8 mmol) and piperidine (8 ml) under N_2_ (bubbler) was heated at 353 K for 2h. The reaction mixture was cooled and evaporated under reduced pressure. The residue was dissolved in a mixture of ethyl acetate (50 ml) and chloro­form (12 mL) and the resulting solution was washed sequentially with water (3 × 100ml) and brine (30 ml) and then dried (MgSO_4_) and evaporated under reduced pressure. The residual dark-purple solid (405 mg) was purified by chromatography over silica gel. Elution with 0–20% ethyl acetate in di­chloro­methane afforded the title compound (328 mg, 81%) as very dark purple–navy coloured blocks (Fig. 7 ▸) after slow evaporation from di­chloro­methane/ethyl acetate, m.p. 463.5–464.5 K (found: *M* ^+^ 482.2683; C_30_H_34_N_4_O_2_ requires *M* ^+^ 482.2676). ^1^H NMR (CDCl_3_, 400 MHz) δ 8.22 (2H, *m*, ArH), 7.70 (2H, *m*, ArH), 3.31 (8H, *m*, 4 × CH_2_N), 3.06 (4H, *q*, *J* = 7.4Hz, 2 × CH_2_Ar), 1.90–1.75 (12H, 2 × CH_2_CH_2_CH_2_), 1.46 (6H, *t*, *J* = 7.4Hz, 2 × CH_3_); ^13^C NMR (CDCl_3_, 100 MHz) δ 183.04, 155.05, 147.52, 140.91, 135.47, 132.72, 126.19, 122.37, 54.93, 28.01, 26.97, 24.72, 12.04.

## Refinement   

Crystal data, data collection and structure refinement details are summarized in Table 3 ▸. Hydrogen atoms potentially involved in hydrogen-bonding inter­actions were located by difference methods and were freely refined. Other H atoms were included in the refinement at calculated positions with C—H = 0.95–0.99 Å and treated as riding with *U*_iso_(H) = 1.2*U*_eq_(C) or 1.52*U*_eq_(O or methyl C). Electron density associated with additional solvent mol­ecules disordered about a fourfold axis was accounted for using the SQUEEZE procedure in *PLATON* (Spek, 2015 ▸).

## Supplementary Material

Crystal structure: contains datablock(s) II, IV, global. DOI: 10.1107/S2056989017009641/zs2382sup1.cif

Structure factors: contains datablock(s) II. DOI: 10.1107/S2056989017009641/zs2382IIsup2.hkl

Structure factors: contains datablock(s) IV. DOI: 10.1107/S2056989017009641/zs2382IVsup3.hkl

Supporting information file. DOI: 10.1107/S2056989017009641/zs2382IIsup4.cml

Supporting information file. DOI: 10.1107/S2056989017009641/zs2382IVsup5.cml

CCDC references: 1559404, 1559403

Additional supporting information:  crystallographic information; 3D view; checkCIF report

## Figures and Tables

**Table 1:** Hydrogen-bond geometry (Å, °) for (II)

*D*—H⋯*A*	*D*—H	H⋯*A*	*D*⋯*A*	*D*—H⋯*A*
O1—H1⋯O2	0.97 (3)	1.62 (3)	2.5270 (16)	155 (2)
O3—H3⋯O4	1.00 (3)	1.58 (3)	2.5225 (17)	154 (2)
C8—H8⋯O1^i^	0.95	2.57	3.227 (2)	126
C19—H19*A*⋯O4^ii^	0.99	2.59	3.418 (2)	142

Symmetry codes: (i) 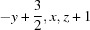; (ii) 
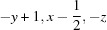
.

**Table 2:** Hydrogen-bond geometry (Å, °) for (IV)

*D*—H⋯*A*	*D*—H	H⋯*A*	*D*⋯*A*	*D*—H⋯*A*
C56—H56*A*⋯O3^i^	0.99	2.54	3.1765 (19)	122

Symmetry code: (i) 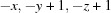.

**Table 3:** Experimental details

	(II)	(IV)
Crystal data		
Chemical formula	C_20_H_16_N_2_O_4_	C_30_H_34_N_4_O_2_
*M*_r_	348.35	482.61
Crystal system, space group	Tetragonal, *P*4/*n*	Triclinic, *P* 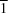
Temperature (K)	123	123
*a*, *b*, *c* (Å)	28.2529 (11), 28.2529 (11), 4.2504 (3)	11.6144 (6), 11.8249 (5), 19.0526 (9)
α, β, γ (°)	90, 90, 90	75.102 (2), 77.310 (2), 83.321 (2)
*V* (Å^3^)	3392.8 (4)	2462.0 (2)
*Z*	8	4
Radiation type	Cu *K*α	Mo *K*α
μ (mm^−1^)	0.80	0.08
Crystal size (mm)	0.25 × 0.04 × 0.04	0.25 × 0.15 × 0.06

Data collection		
Diffractometer	Oxford Gemini Ultra CCD	Bruker APEXII CCD
Absorption correction	Multi-scan (*CrysAlis PRO*; Rigaku OD, 2015 ▸)	Multi-scan (*SADABS*; Bruker, 2014 ▸)
*T*_min_, *T*_max_	0.857, 1.000	0.708, 0.746
No. of measured, independent and observed [*I* > 2σ(*I*)] reflections	10284, 2986, 2391	46624, 11784, 7969
*R*_int_	0.028	0.046
(sin θ/λ)_max_ (Å^−1^)	0.596	0.660

Refinement		
*R*[*F* ^2^ > 2σ(*F* ^2^)], *wR*(*F* ^2^), *S*	0.038, 0.105, 1.03	0.047, 0.113, 1.03
No. of reflections	2986	11784
No. of parameters	243	653
H-atom treatment	H atoms treated by a mixture of independent and constrained refinement	H-atom parameters constrained
Δρ_max_, Δρ_min_ (e Å^−3^)	0.16, −0.19	0.26, −0.23

Computer programs: *CrysAlis PRO* (Rigaku OD, 2015 ▸), *APEX2* (and *SAINT* (Bruker, 2014 ▸), *SHELXT* (Sheldrick, 2015*a* ▸), *SHELXL2016* (Sheldrick, 2015*b* ▸), *X-SEED* (Barbour, 2001 ▸) and *publCIF* (Westrip, 2010 ▸).

## supplementary crystallographic information

### 2,3-Diethyl-5,12-dihydroxynaphtho[2,3-g]quinoxaline-6,11-dione (II) Crystal data

**Table:**

C_20_H_16_N_2_O_4_	Melting point = 507–509 K
*M**_r_* = 348.35	Cu *K*α radiation, λ = 1.54184 Å
Tetragonal, *P*4/*n*	Cell parameters from 2972 reflections
*a* = 28.2529 (11) Å	θ = 4.4–66.9°
*c* = 4.2504 (3) Å	µ = 0.80 mm^−^^1^
*V* = 3392.8 (4) Å^3^	*T* = 123 K
*Z* = 8	Needle, orange
*F*(000) = 1456	0.25 × 0.04 × 0.04 mm
*D*_x_ = 1.364 Mg m^−^^3^	

### 2,3-Diethyl-5,12-dihydroxynaphtho[2,3-g]quinoxaline-6,11-dione (II) Data collection

**Table:**

Oxford Gemini Ultra CCD diffractometer	2986 independent reflections
Radiation source: fine focus sealed tube	2391 reflections with *I* > 2σ(*I*)
Detector resolution: 10.3389 pixels mm^-1^	*R*_int_ = 0.028
ω scans	θ_max_ = 66.7°, θ_min_ = 5.0°
Absorption correction: multi-scan (CrysAlis PRO; Rigaku OD, 2015)	*h* = −32→32
*T*_min_ = 0.857, *T*_max_ = 1.000	*k* = −30→33
10284 measured reflections	*l* = −4→5

### 2,3-Diethyl-5,12-dihydroxynaphtho[2,3-g]quinoxaline-6,11-dione (II) Refinement

**Table:**

Refinement on *F*^2^	0 restraints
Least-squares matrix: full	Hydrogen site location: mixed
*R*[*F*^2^ > 2σ(*F*^2^)] = 0.038	H atoms treated by a mixture of independent and constrained refinement
*wR*(*F*^2^) = 0.105	*w* = 1/[σ^2^(*F*_o_^2^) + (0.0477*P*)^2^ + 1.2563*P*] where *P* = (*F*_o_^2^ + 2*F*_c_^2^)/3
*S* = 1.03	(Δ/σ)_max_ = 0.001
2986 reflections	Δρ_max_ = 0.16 e Å^−^^3^
243 parameters	Δρ_min_ = −0.19 e Å^−^^3^

### 2,3-Diethyl-5,12-dihydroxynaphtho[2,3-g]quinoxaline-6,11-dione (II) Special details

**Table:**

Geometry. All esds (except the esd in the dihedral angle between two l.s. planes) are estimated using the full covariance matrix. The cell esds are taken into account individually in the estimation of esds in distances, angles and torsion angles; correlations between esds in cell parameters are only used when they are defined by crystal symmetry. An approximate (isotropic) treatment of cell esds is used for estimating esds involving l.s. planes.
Refinement. Disordered solvent molecules were accounted for using PLATON SQUEEZE (Spek, 2015).

### 2,3-Diethyl-5,12-dihydroxynaphtho[2,3-g]quinoxaline-6,11-dione (II) Fractional atomic coordinates and isotropic or equivalent isotropic displacement parameters (Å^2^)

**Table:**

	*x*	*y*	*z*	*U*_iso_*/*U*_eq_	
O1	0.67025 (4)	0.54392 (4)	0.1718 (3)	0.0328 (3)	
O2	0.72757 (4)	0.59355 (4)	0.4871 (3)	0.0390 (3)	
O3	0.81968 (4)	0.43226 (4)	−0.2039 (3)	0.0378 (3)	
O4	0.87451 (4)	0.48403 (4)	0.1138 (3)	0.0379 (3)	
N1	0.65539 (4)	0.47227 (4)	−0.2404 (3)	0.0290 (3)	
N2	0.73105 (4)	0.41228 (4)	−0.4052 (3)	0.0301 (3)	
C1	0.69990 (5)	0.47958 (5)	−0.1266 (4)	0.0266 (3)	
C2	0.70777 (5)	0.51838 (5)	0.0856 (4)	0.0273 (3)	
C3	0.75334 (5)	0.52791 (5)	0.1904 (4)	0.0272 (3)	
C4	0.76120 (5)	0.56822 (5)	0.3970 (4)	0.0300 (4)	
C5	0.81005 (5)	0.57931 (6)	0.4958 (4)	0.0307 (4)	
C6	0.81855 (6)	0.61881 (6)	0.6840 (4)	0.0368 (4)	
H6	0.792925	0.638278	0.748691	0.044*	
C7	0.86416 (6)	0.62974 (6)	0.7769 (4)	0.0397 (4)	
H7	0.869763	0.656889	0.903442	0.048*	
C8	0.90185 (6)	0.60122 (6)	0.6862 (4)	0.0391 (4)	
H8	0.933073	0.608708	0.752457	0.047*	
C9	0.89390 (6)	0.56199 (6)	0.4997 (4)	0.0357 (4)	
H9	0.919743	0.542627	0.437336	0.043*	
C10	0.84809 (6)	0.55063 (6)	0.4023 (4)	0.0312 (4)	
C11	0.84036 (5)	0.50917 (5)	0.1971 (4)	0.0307 (4)	
C12	0.79233 (5)	0.49866 (5)	0.0926 (4)	0.0273 (3)	
C13	0.78477 (5)	0.46048 (5)	−0.1055 (4)	0.0287 (3)	
C14	0.73774 (5)	0.45040 (5)	−0.2159 (4)	0.0276 (3)	
C15	0.68771 (5)	0.40428 (5)	−0.5072 (4)	0.0301 (4)	
C16	0.64927 (5)	0.43592 (5)	−0.4317 (4)	0.0295 (4)	
C17	0.67933 (6)	0.36048 (6)	−0.7008 (4)	0.0357 (4)	
H17A	0.665387	0.369903	−0.905126	0.043*	
H17B	0.655945	0.340279	−0.591035	0.043*	
C18	0.72365 (6)	0.33147 (6)	−0.7628 (5)	0.0415 (4)	
H18A	0.715472	0.303671	−0.889563	0.062*	
H18B	0.746756	0.350852	−0.876749	0.062*	
H18C	0.737301	0.321187	−0.562219	0.062*	
C19	0.60067 (6)	0.42782 (6)	−0.5658 (4)	0.0344 (4)	
H19A	0.586627	0.399785	−0.461847	0.041*	
H19B	0.603839	0.420419	−0.792472	0.041*	
C20	0.56686 (6)	0.46916 (7)	−0.5296 (5)	0.0447 (5)	
H20A	0.536206	0.460944	−0.622739	0.067*	
H20B	0.562632	0.476279	−0.305694	0.067*	
H20C	0.579878	0.496953	−0.636958	0.067*	
H1	0.6842 (9)	0.5668 (9)	0.314 (6)	0.073 (7)*	
H3	0.8478 (9)	0.4458 (9)	−0.091 (6)	0.072 (7)*	

### 2,3-Diethyl-5,12-dihydroxynaphtho[2,3-g]quinoxaline-6,11-dione (II) Atomic displacement parameters (Å^2^)

**Table:**

	*U*^11^	*U*^22^	*U*^33^	*U*^12^	*U*^13^	*U*^23^
O1	0.0264 (6)	0.0316 (6)	0.0405 (6)	0.0052 (5)	0.0000 (5)	−0.0013 (5)
O2	0.0321 (6)	0.0370 (6)	0.0479 (7)	0.0048 (5)	−0.0002 (5)	−0.0077 (5)
O3	0.0261 (6)	0.0361 (6)	0.0511 (7)	0.0051 (5)	0.0015 (5)	−0.0069 (6)
O4	0.0251 (6)	0.0375 (6)	0.0511 (7)	0.0029 (5)	0.0002 (5)	−0.0003 (5)
N1	0.0264 (7)	0.0281 (7)	0.0326 (7)	−0.0013 (5)	0.0009 (5)	0.0055 (6)
N2	0.0288 (7)	0.0292 (7)	0.0323 (7)	−0.0012 (5)	0.0018 (6)	0.0029 (6)
C1	0.0245 (7)	0.0262 (7)	0.0291 (8)	−0.0006 (6)	0.0011 (6)	0.0073 (6)
C2	0.0257 (8)	0.0253 (7)	0.0308 (8)	0.0030 (6)	0.0033 (6)	0.0065 (6)
C3	0.0263 (8)	0.0259 (7)	0.0296 (8)	0.0007 (6)	0.0021 (6)	0.0069 (6)
C4	0.0311 (8)	0.0281 (8)	0.0309 (8)	0.0006 (7)	0.0011 (7)	0.0052 (7)
C5	0.0305 (8)	0.0299 (8)	0.0315 (8)	−0.0033 (6)	−0.0008 (7)	0.0060 (7)
C6	0.0381 (9)	0.0332 (8)	0.0390 (9)	−0.0023 (7)	0.0004 (7)	0.0018 (7)
C7	0.0426 (10)	0.0362 (9)	0.0403 (10)	−0.0083 (7)	−0.0037 (8)	0.0007 (8)
C8	0.0341 (9)	0.0406 (9)	0.0424 (10)	−0.0086 (7)	−0.0045 (8)	0.0062 (8)
C9	0.0304 (8)	0.0366 (9)	0.0401 (10)	−0.0031 (7)	−0.0019 (7)	0.0068 (8)
C10	0.0295 (8)	0.0309 (8)	0.0333 (9)	−0.0037 (7)	0.0000 (7)	0.0075 (7)
C11	0.0280 (8)	0.0294 (8)	0.0347 (9)	0.0001 (6)	0.0012 (7)	0.0066 (7)
C12	0.0243 (7)	0.0265 (7)	0.0312 (8)	0.0006 (6)	0.0014 (6)	0.0070 (6)
C13	0.0249 (7)	0.0277 (8)	0.0334 (8)	0.0023 (6)	0.0029 (6)	0.0054 (7)
C14	0.0278 (8)	0.0258 (7)	0.0292 (8)	0.0004 (6)	0.0028 (6)	0.0055 (6)
C15	0.0311 (8)	0.0294 (8)	0.0299 (8)	−0.0011 (6)	0.0020 (7)	0.0051 (6)
C16	0.0293 (8)	0.0277 (8)	0.0317 (8)	−0.0023 (6)	0.0018 (6)	0.0053 (7)
C17	0.0350 (9)	0.0345 (9)	0.0377 (9)	−0.0014 (7)	−0.0016 (7)	−0.0014 (7)
C18	0.0426 (10)	0.0349 (9)	0.0470 (11)	0.0005 (8)	−0.0002 (8)	−0.0077 (8)
C19	0.0284 (8)	0.0358 (9)	0.0389 (9)	−0.0039 (7)	−0.0008 (7)	0.0011 (7)
C20	0.0308 (9)	0.0432 (10)	0.0602 (12)	0.0003 (8)	−0.0079 (8)	−0.0035 (9)

### 2,3-Diethyl-5,12-dihydroxynaphtho[2,3-g]quinoxaline-6,11-dione (II) Geometric parameters (Å, º)

**Table:**

O1—C2	1.3333 (18)	C8—H8	0.9500
O1—H1	0.97 (3)	C9—C10	1.396 (2)
O2—C4	1.2495 (19)	C9—H9	0.9500
O3—C13	1.3354 (19)	C10—C11	1.477 (2)
O3—H3	1.00 (3)	C11—C12	1.458 (2)
O4—C11	1.2493 (19)	C12—C13	1.385 (2)
N1—C16	1.321 (2)	C13—C14	1.438 (2)
N1—C1	1.363 (2)	C15—C16	1.443 (2)
N2—C15	1.318 (2)	C15—C17	1.505 (2)
N2—C14	1.358 (2)	C16—C19	1.504 (2)
C1—C14	1.402 (2)	C17—C18	1.519 (2)
C1—C2	1.437 (2)	C17—H17A	0.9900
C2—C3	1.389 (2)	C17—H17B	0.9900
C3—C12	1.438 (2)	C18—H18A	0.9800
C3—C4	1.455 (2)	C18—H18B	0.9800
C4—C5	1.476 (2)	C18—H18C	0.9800
C5—C6	1.394 (2)	C19—C20	1.517 (2)
C5—C10	1.403 (2)	C19—H19A	0.9900
C6—C7	1.383 (2)	C19—H19B	0.9900
C6—H6	0.9500	C20—H20A	0.9800
C7—C8	1.390 (3)	C20—H20B	0.9800
C7—H7	0.9500	C20—H20C	0.9800
C8—C9	1.381 (3)		
			
C2—O1—H1	102.1 (14)	C13—C12—C11	119.16 (14)
C13—O3—H3	102.0 (14)	C3—C12—C11	120.51 (14)
C16—N1—C1	117.19 (13)	O3—C13—C12	122.78 (14)
C15—N2—C14	117.39 (14)	O3—C13—C14	117.52 (14)
N1—C1—C14	121.20 (14)	C12—C13—C14	119.69 (14)
N1—C1—C2	118.75 (13)	N2—C14—C1	121.37 (14)
C14—C1—C2	120.04 (13)	N2—C14—C13	118.65 (13)
O1—C2—C3	122.97 (14)	C1—C14—C13	119.98 (14)
O1—C2—C1	117.53 (13)	N2—C15—C16	121.32 (15)
C3—C2—C1	119.50 (14)	N2—C15—C17	117.81 (14)
C2—C3—C12	120.41 (14)	C16—C15—C17	120.86 (14)
C2—C3—C4	119.12 (14)	N1—C16—C15	121.34 (14)
C12—C3—C4	120.46 (13)	N1—C16—C19	118.09 (14)
O2—C4—C3	121.14 (14)	C15—C16—C19	120.56 (14)
O2—C4—C5	120.14 (14)	C15—C17—C18	114.13 (14)
C3—C4—C5	118.71 (14)	C15—C17—H17A	108.7
C6—C5—C10	119.52 (15)	C18—C17—H17A	108.7
C6—C5—C4	119.61 (15)	C15—C17—H17B	108.7
C10—C5—C4	120.86 (14)	C18—C17—H17B	108.7
C7—C6—C5	120.23 (16)	H17A—C17—H17B	107.6
C7—C6—H6	119.9	C17—C18—H18A	109.5
C5—C6—H6	119.9	C17—C18—H18B	109.5
C6—C7—C8	120.34 (17)	H18A—C18—H18B	109.5
C6—C7—H7	119.8	C17—C18—H18C	109.5
C8—C7—H7	119.8	H18A—C18—H18C	109.5
C9—C8—C7	120.01 (16)	H18B—C18—H18C	109.5
C9—C8—H8	120.0	C16—C19—C20	114.80 (14)
C7—C8—H8	120.0	C16—C19—H19A	108.6
C8—C9—C10	120.35 (16)	C20—C19—H19A	108.6
C8—C9—H9	119.8	C16—C19—H19B	108.6
C10—C9—H9	119.8	C20—C19—H19B	108.6
C9—C10—C5	119.56 (15)	H19A—C19—H19B	107.5
C9—C10—C11	119.64 (15)	C19—C20—H20A	109.5
C5—C10—C11	120.79 (14)	C19—C20—H20B	109.5
O4—C11—C12	121.09 (15)	H20A—C20—H20B	109.5
O4—C11—C10	120.28 (14)	C19—C20—H20C	109.5
C12—C11—C10	118.62 (14)	H20A—C20—H20C	109.5
C13—C12—C3	120.32 (13)	H20B—C20—H20C	109.5
			
N2—C15—C17—C18	−1.9 (2)	N1—C16—C19—C20	14.3 (2)

### 2,3-Diethyl-5,12-dihydroxynaphtho[2,3-g]quinoxaline-6,11-dione (II) Hydrogen-bond geometry (Å, º)

**Table:**

*D*—H···*A*	*D*—H	H···*A*	*D*···*A*	*D*—H···*A*
O1—H1···O2	0.97 (3)	1.62 (3)	2.5270 (16)	155 (2)
O3—H3···O4	1.00 (3)	1.58 (3)	2.5225 (17)	154 (2)
C8—H8···O1^i^	0.95	2.57	3.227 (2)	126
C19—H19*A*···O4^ii^	0.99	2.59	3.418 (2)	142

Symmetry codes: (i) −*y*+3/2, *x*, *z*+1; (ii) −*y*+1, *x*−1/2, −*z*.

### 2,3-Diethyl-5,12-bis(piperidin-1-yl)naphtho[2,3-g]quinoxaline-6,11-dione (IV) Crystal data

**Table:**

C_30_H_34_N_4_O_2_	*F*(000) = 1032
*M**_r_* = 482.61	*D*_x_ = 1.302 Mg m^−^^3^
Triclinic, *P*1	Melting point = 463.5–464.5 K
*a* = 11.6144 (6) Å	Mo *K*α radiation, λ = 0.71073 Å
*b* = 11.8249 (5) Å	Cell parameters from 8449 reflections
*c* = 19.0526 (9) Å	θ = 2.3–26.6°
α = 75.102 (2)°	µ = 0.08 mm^−^^1^
β = 77.310 (2)°	*T* = 123 K
γ = 83.321 (2)°	Prismatic, dark red
*V* = 2462.0 (2) Å^3^	0.25 × 0.15 × 0.06 mm
*Z* = 4	

### 2,3-Diethyl-5,12-bis(piperidin-1-yl)naphtho[2,3-g]quinoxaline-6,11-dione (IV) Data collection

**Table:**

Bruker APEXII CCD diffractometer	7969 reflections with *I* > 2σ(*I*)
Radiation source: fine focus sealed tube	*R*_int_ = 0.046
ω scans	θ_max_ = 28.0°, θ_min_ = 1.1°
Absorption correction: multi-scan (SADABS; Bruker, 2014)	*h* = −15→12
*T*_min_ = 0.708, *T*_max_ = 0.746	*k* = −15→13
46624 measured reflections	*l* = −25→25
11784 independent reflections	

### 2,3-Diethyl-5,12-bis(piperidin-1-yl)naphtho[2,3-g]quinoxaline-6,11-dione (IV) Refinement

**Table:**

Refinement on *F*^2^	0 restraints
Least-squares matrix: full	Hydrogen site location: inferred from neighbouring sites
*R*[*F*^2^ > 2σ(*F*^2^)] = 0.047	H-atom parameters constrained
*wR*(*F*^2^) = 0.113	*w* = 1/[σ^2^(*F*_o_^2^) + (0.0456*P*)^2^ + 0.481*P*] where *P* = (*F*_o_^2^ + 2*F*_c_^2^)/3
*S* = 1.03	(Δ/σ)_max_ < 0.001
11784 reflections	Δρ_max_ = 0.26 e Å^−^^3^
653 parameters	Δρ_min_ = −0.23 e Å^−^^3^

### 2,3-Diethyl-5,12-bis(piperidin-1-yl)naphtho[2,3-g]quinoxaline-6,11-dione (IV) Special details

**Table:**

Geometry. All esds (except the esd in the dihedral angle between two l.s. planes) are estimated using the full covariance matrix. The cell esds are taken into account individually in the estimation of esds in distances, angles and torsion angles; correlations between esds in cell parameters are only used when they are defined by crystal symmetry. An approximate (isotropic) treatment of cell esds is used for estimating esds involving l.s. planes.

### 2,3-Diethyl-5,12-bis(piperidin-1-yl)naphtho[2,3-g]quinoxaline-6,11-dione (IV) Fractional atomic coordinates and isotropic or equivalent isotropic displacement parameters (Å^2^)

**Table:**

	*x*	*y*	*z*	*U*_iso_*/*U*_eq_	
O1	0.64307 (10)	0.76179 (9)	0.10075 (6)	0.0291 (3)	
O2	0.44074 (9)	0.37783 (9)	0.09799 (6)	0.0227 (2)	
O3	0.00984 (9)	0.43530 (9)	0.39662 (6)	0.0238 (3)	
O4	0.20118 (9)	0.83312 (9)	0.38244 (6)	0.0244 (3)	
N1	0.92806 (11)	0.56473 (11)	−0.08692 (7)	0.0201 (3)	
N2	0.87671 (11)	0.33134 (10)	−0.02637 (7)	0.0193 (3)	
N3	0.76849 (11)	0.74582 (10)	−0.04703 (7)	0.0204 (3)	
N4	0.67498 (11)	0.27048 (10)	0.08042 (7)	0.0194 (3)	
N5	0.35733 (11)	0.31461 (10)	0.53063 (7)	0.0178 (3)	
N6	0.36271 (11)	0.51220 (10)	0.58404 (7)	0.0182 (3)	
N7	0.23683 (11)	0.32525 (10)	0.41697 (7)	0.0178 (3)	
N8	0.23239 (11)	0.71992 (10)	0.53315 (7)	0.0178 (3)	
C1	0.82928 (13)	0.53690 (13)	−0.03378 (8)	0.0177 (3)	
C2	0.75273 (13)	0.63060 (12)	−0.00901 (8)	0.0177 (3)	
C3	0.65573 (13)	0.59826 (12)	0.04761 (8)	0.0171 (3)	
C4	0.59410 (14)	0.68035 (13)	0.09244 (8)	0.0206 (3)	
C5	0.46967 (14)	0.65994 (13)	0.13028 (8)	0.0194 (3)	
C6	0.40230 (15)	0.74326 (13)	0.16528 (8)	0.0247 (4)	
H6	0.437196	0.811465	0.166282	0.030*	
C7	0.28514 (15)	0.72663 (14)	0.19840 (9)	0.0281 (4)	
H7	0.239989	0.782887	0.222699	0.034*	
C8	0.23321 (15)	0.62768 (14)	0.19622 (9)	0.0262 (4)	
H8	0.152230	0.617286	0.218220	0.031*	
C9	0.29892 (14)	0.54455 (13)	0.16220 (8)	0.0216 (3)	
H9	0.263028	0.477133	0.160859	0.026*	
C10	0.41767 (13)	0.55915 (13)	0.12984 (8)	0.0179 (3)	
C11	0.49024 (13)	0.46378 (12)	0.09913 (8)	0.0177 (3)	
C12	0.61682 (13)	0.47950 (12)	0.06835 (8)	0.0171 (3)	
C13	0.69338 (13)	0.38800 (12)	0.04854 (8)	0.0165 (3)	
C14	0.80214 (13)	0.41920 (12)	−0.00387 (8)	0.0168 (3)	
C15	0.97083 (13)	0.36001 (13)	−0.07839 (8)	0.0198 (3)	
C16	0.99814 (13)	0.47940 (13)	−0.10873 (8)	0.0210 (3)	
C17	1.04835 (14)	0.26396 (13)	−0.10759 (9)	0.0255 (4)	
H17A	1.122545	0.252641	−0.088430	0.031*	
H17B	1.069489	0.289889	−0.162262	0.031*	
C18	0.99291 (15)	0.14705 (14)	−0.08728 (10)	0.0304 (4)	
H18A	0.981807	0.114987	−0.033604	0.046*	
H18B	1.044970	0.092437	−0.112800	0.046*	
H18C	0.916122	0.158141	−0.102318	0.046*	
C19	1.10924 (14)	0.51096 (14)	−0.16537 (9)	0.0286 (4)	
H19A	1.104599	0.487061	−0.210757	0.034*	
H19B	1.177392	0.465543	−0.145914	0.034*	
C20	1.13193 (15)	0.64004 (15)	−0.18601 (10)	0.0325 (4)	
H20A	1.069701	0.685231	−0.210746	0.049*	
H20B	1.208913	0.652470	−0.219542	0.049*	
H20C	1.131877	0.665951	−0.141106	0.049*	
C21	0.66797 (14)	0.83081 (13)	−0.05500 (9)	0.0239 (4)	
H21A	0.663393	0.883897	−0.021748	0.029*	
H21B	0.593930	0.789359	−0.040503	0.029*	
C22	0.68064 (15)	0.90205 (13)	−0.13465 (9)	0.0282 (4)	
H22A	0.674677	0.849979	−0.166756	0.034*	
H22B	0.614849	0.963176	−0.138234	0.034*	
C23	0.79740 (15)	0.96007 (14)	−0.16248 (10)	0.0330 (4)	
H23A	0.798951	1.021122	−0.135629	0.040*	
H23B	0.805829	0.998324	−0.215905	0.040*	
C24	0.89973 (16)	0.86857 (14)	−0.15035 (10)	0.0321 (4)	
H24A	0.904586	0.813589	−0.182426	0.039*	
H24B	0.975021	0.907830	−0.164113	0.039*	
C25	0.88176 (14)	0.80104 (13)	−0.06978 (9)	0.0242 (4)	
H25A	0.947023	0.739996	−0.063018	0.029*	
H25B	0.883470	0.855131	−0.038113	0.029*	
C26	0.61434 (14)	0.22997 (13)	0.15705 (8)	0.0226 (3)	
H26A	0.531105	0.216848	0.158534	0.027*	
H26B	0.614563	0.290403	0.184600	0.027*	
C27	0.67639 (15)	0.11606 (13)	0.19334 (9)	0.0265 (4)	
H27A	0.757300	0.131197	0.195810	0.032*	
H27B	0.632714	0.086958	0.244615	0.032*	
C28	0.68319 (16)	0.02295 (13)	0.14990 (9)	0.0290 (4)	
H28A	0.729186	−0.047971	0.172085	0.035*	
H28B	0.602556	0.000766	0.152299	0.035*	
C29	0.74206 (15)	0.07018 (13)	0.06962 (9)	0.0268 (4)	
H29A	0.740326	0.011871	0.040792	0.032*	
H29B	0.825800	0.083003	0.066931	0.032*	
C30	0.67921 (14)	0.18493 (13)	0.03607 (9)	0.0213 (3)	
H30A	0.721546	0.216577	−0.015215	0.026*	
H30B	0.597765	0.170718	0.034117	0.026*	
C31	0.29857 (13)	0.41777 (12)	0.50286 (8)	0.0161 (3)	
C32	0.22838 (13)	0.41992 (12)	0.44827 (8)	0.0158 (3)	
C33	0.16162 (13)	0.52427 (12)	0.42530 (8)	0.0159 (3)	
C34	0.05634 (13)	0.52508 (12)	0.39413 (8)	0.0168 (3)	
C35	−0.00127 (13)	0.64074 (12)	0.36195 (8)	0.0164 (3)	
C36	−0.09976 (13)	0.64368 (13)	0.33112 (8)	0.0195 (3)	
H36	−0.131580	0.572516	0.332467	0.023*	
C37	−0.15175 (14)	0.75005 (13)	0.29841 (8)	0.0219 (3)	
H37	−0.219112	0.751708	0.277412	0.026*	
C38	−0.10530 (14)	0.85434 (13)	0.29632 (9)	0.0241 (4)	
H38	−0.140896	0.927147	0.273718	0.029*	
C39	−0.00771 (14)	0.85251 (13)	0.32695 (8)	0.0213 (3)	
H39	0.023522	0.923999	0.325567	0.026*	
C40	0.04521 (13)	0.74559 (12)	0.36001 (8)	0.0173 (3)	
C41	0.14774 (13)	0.74399 (12)	0.39516 (8)	0.0173 (3)	
C42	0.18326 (13)	0.63122 (12)	0.44271 (8)	0.0162 (3)	
C43	0.24231 (13)	0.62819 (12)	0.49935 (8)	0.0163 (3)	
C44	0.30259 (12)	0.51778 (12)	0.52920 (8)	0.0159 (3)	
C45	0.41745 (13)	0.41175 (13)	0.61117 (8)	0.0186 (3)	
C46	0.41327 (13)	0.30999 (13)	0.58443 (8)	0.0176 (3)	
C47	0.48628 (15)	0.40615 (14)	0.67057 (9)	0.0259 (4)	
H47A	0.568872	0.378011	0.653634	0.031*	
H47B	0.452188	0.347711	0.715611	0.031*	
C48	0.48767 (16)	0.52105 (14)	0.69094 (10)	0.0308 (4)	
H48A	0.517334	0.580874	0.646329	0.046*	
H48B	0.539328	0.511346	0.726689	0.046*	
H48C	0.407264	0.545562	0.713026	0.046*	
C49	0.47002 (14)	0.19372 (12)	0.61958 (9)	0.0213 (3)	
H49A	0.448772	0.181559	0.674117	0.026*	
H49B	0.557021	0.196911	0.604884	0.026*	
C50	0.43381 (14)	0.08951 (13)	0.59841 (9)	0.0239 (4)	
H50A	0.347793	0.085241	0.612977	0.036*	
H50B	0.472438	0.017246	0.623895	0.036*	
H50C	0.457852	0.098836	0.544718	0.036*	
C51	0.22785 (15)	0.34127 (13)	0.33992 (8)	0.0231 (4)	
H51A	0.243904	0.422966	0.312547	0.028*	
H51B	0.146626	0.327201	0.337434	0.028*	
C52	0.31617 (15)	0.25666 (13)	0.30397 (9)	0.0260 (4)	
H52A	0.306884	0.266219	0.252305	0.031*	
H52B	0.397673	0.275183	0.302702	0.031*	
C53	0.29684 (15)	0.13058 (13)	0.34685 (9)	0.0278 (4)	
H53A	0.358319	0.077208	0.325065	0.033*	
H53B	0.218614	0.108973	0.343697	0.033*	
C54	0.30284 (15)	0.11791 (13)	0.42761 (9)	0.0252 (4)	
H54A	0.284564	0.037295	0.456246	0.030*	
H54B	0.383995	0.130809	0.430914	0.030*	
C55	0.21586 (14)	0.20538 (12)	0.46088 (8)	0.0194 (3)	
H55A	0.134086	0.187461	0.462318	0.023*	
H55B	0.224604	0.198662	0.512350	0.023*	
C56	0.12623 (13)	0.79869 (13)	0.53730 (9)	0.0200 (3)	
H56A	0.060834	0.762672	0.527024	0.024*	
H56B	0.141143	0.873316	0.499566	0.024*	
C57	0.09159 (14)	0.82268 (14)	0.61428 (9)	0.0242 (4)	
H57A	0.021908	0.879459	0.616046	0.029*	
H57B	0.069186	0.748964	0.651245	0.029*	
C58	0.19249 (14)	0.87157 (14)	0.63418 (9)	0.0257 (4)	
H58A	0.170315	0.878707	0.686160	0.031*	
H58B	0.207459	0.950705	0.601825	0.031*	
C59	0.30456 (14)	0.79096 (14)	0.62466 (9)	0.0245 (4)	
H59A	0.371551	0.828007	0.632284	0.029*	
H59B	0.293493	0.715943	0.662497	0.029*	
C60	0.33318 (13)	0.76704 (13)	0.54812 (8)	0.0190 (3)	
H60A	0.353546	0.840682	0.510516	0.023*	
H60B	0.402596	0.710193	0.544475	0.023*	

### 2,3-Diethyl-5,12-bis(piperidin-1-yl)naphtho[2,3-g]quinoxaline-6,11-dione (IV) Atomic displacement parameters (Å^2^)

**Table:**

	*U*^11^	*U*^22^	*U*^33^	*U*^12^	*U*^13^	*U*^23^
O1	0.0291 (7)	0.0264 (6)	0.0372 (7)	−0.0038 (5)	−0.0057 (5)	−0.0170 (5)
O2	0.0200 (6)	0.0229 (6)	0.0277 (6)	−0.0031 (5)	−0.0056 (5)	−0.0087 (5)
O3	0.0232 (6)	0.0181 (5)	0.0323 (6)	−0.0030 (5)	−0.0090 (5)	−0.0062 (5)
O4	0.0289 (6)	0.0161 (5)	0.0281 (6)	−0.0050 (5)	−0.0082 (5)	−0.0014 (5)
N1	0.0179 (7)	0.0234 (7)	0.0184 (7)	−0.0019 (5)	−0.0028 (5)	−0.0041 (5)
N2	0.0182 (7)	0.0202 (7)	0.0199 (7)	0.0038 (5)	−0.0059 (6)	−0.0058 (5)
N3	0.0185 (7)	0.0164 (6)	0.0263 (7)	−0.0030 (5)	−0.0049 (6)	−0.0041 (6)
N4	0.0223 (7)	0.0152 (6)	0.0193 (7)	−0.0009 (5)	−0.0018 (6)	−0.0036 (5)
N5	0.0168 (7)	0.0171 (6)	0.0175 (7)	0.0011 (5)	−0.0026 (5)	−0.0021 (5)
N6	0.0180 (7)	0.0190 (6)	0.0180 (7)	−0.0017 (5)	−0.0047 (5)	−0.0039 (5)
N7	0.0248 (7)	0.0126 (6)	0.0172 (6)	0.0009 (5)	−0.0066 (6)	−0.0043 (5)
N8	0.0160 (7)	0.0162 (6)	0.0240 (7)	−0.0005 (5)	−0.0055 (5)	−0.0084 (5)
C1	0.0167 (8)	0.0207 (8)	0.0165 (8)	−0.0005 (6)	−0.0056 (6)	−0.0042 (6)
C2	0.0182 (8)	0.0176 (8)	0.0194 (8)	−0.0018 (6)	−0.0076 (6)	−0.0045 (6)
C3	0.0172 (8)	0.0161 (7)	0.0190 (8)	0.0012 (6)	−0.0059 (6)	−0.0051 (6)
C4	0.0238 (9)	0.0169 (8)	0.0215 (8)	0.0007 (6)	−0.0071 (7)	−0.0038 (6)
C5	0.0232 (8)	0.0190 (8)	0.0152 (7)	0.0035 (6)	−0.0052 (6)	−0.0034 (6)
C6	0.0324 (10)	0.0201 (8)	0.0196 (8)	0.0038 (7)	−0.0040 (7)	−0.0046 (7)
C7	0.0311 (10)	0.0246 (9)	0.0227 (9)	0.0116 (7)	−0.0005 (7)	−0.0051 (7)
C8	0.0207 (9)	0.0312 (9)	0.0196 (8)	0.0072 (7)	−0.0004 (7)	−0.0002 (7)
C9	0.0210 (8)	0.0250 (8)	0.0163 (8)	0.0022 (6)	−0.0050 (7)	−0.0012 (6)
C10	0.0185 (8)	0.0203 (8)	0.0139 (7)	0.0031 (6)	−0.0054 (6)	−0.0021 (6)
C11	0.0198 (8)	0.0187 (8)	0.0144 (7)	−0.0012 (6)	−0.0043 (6)	−0.0030 (6)
C12	0.0173 (8)	0.0185 (7)	0.0159 (7)	−0.0005 (6)	−0.0041 (6)	−0.0044 (6)
C13	0.0167 (8)	0.0171 (7)	0.0167 (7)	−0.0001 (6)	−0.0057 (6)	−0.0040 (6)
C14	0.0159 (8)	0.0183 (7)	0.0166 (7)	0.0023 (6)	−0.0055 (6)	−0.0046 (6)
C15	0.0167 (8)	0.0244 (8)	0.0191 (8)	0.0033 (6)	−0.0059 (7)	−0.0065 (6)
C16	0.0178 (8)	0.0270 (8)	0.0189 (8)	−0.0002 (6)	−0.0045 (7)	−0.0064 (7)
C17	0.0233 (9)	0.0283 (9)	0.0221 (8)	0.0060 (7)	−0.0019 (7)	−0.0066 (7)
C18	0.0283 (10)	0.0278 (9)	0.0374 (10)	0.0078 (7)	−0.0065 (8)	−0.0160 (8)
C19	0.0230 (9)	0.0341 (10)	0.0261 (9)	−0.0025 (7)	0.0004 (7)	−0.0065 (8)
C20	0.0268 (10)	0.0403 (10)	0.0252 (9)	−0.0082 (8)	0.0028 (8)	−0.0027 (8)
C21	0.0222 (9)	0.0164 (8)	0.0346 (9)	0.0000 (6)	−0.0089 (7)	−0.0067 (7)
C22	0.0316 (10)	0.0180 (8)	0.0365 (10)	−0.0015 (7)	−0.0130 (8)	−0.0040 (7)
C23	0.0377 (11)	0.0213 (9)	0.0367 (10)	−0.0067 (8)	−0.0094 (9)	0.0029 (8)
C24	0.0306 (10)	0.0258 (9)	0.0351 (10)	−0.0095 (7)	−0.0032 (8)	0.0019 (8)
C25	0.0234 (9)	0.0201 (8)	0.0315 (9)	−0.0058 (7)	−0.0081 (7)	−0.0063 (7)
C26	0.0253 (9)	0.0199 (8)	0.0212 (8)	−0.0034 (7)	−0.0012 (7)	−0.0044 (7)
C27	0.0325 (10)	0.0209 (8)	0.0241 (9)	−0.0051 (7)	−0.0073 (7)	0.0009 (7)
C28	0.0330 (10)	0.0173 (8)	0.0364 (10)	−0.0027 (7)	−0.0123 (8)	−0.0009 (7)
C29	0.0306 (10)	0.0171 (8)	0.0353 (10)	0.0034 (7)	−0.0097 (8)	−0.0104 (7)
C30	0.0215 (8)	0.0196 (8)	0.0254 (9)	−0.0014 (6)	−0.0062 (7)	−0.0088 (7)
C31	0.0153 (8)	0.0158 (7)	0.0155 (7)	−0.0001 (6)	−0.0006 (6)	−0.0033 (6)
C32	0.0169 (8)	0.0142 (7)	0.0151 (7)	−0.0013 (6)	−0.0006 (6)	−0.0036 (6)
C33	0.0176 (8)	0.0152 (7)	0.0146 (7)	−0.0009 (6)	−0.0025 (6)	−0.0036 (6)
C34	0.0174 (8)	0.0168 (7)	0.0157 (7)	−0.0013 (6)	−0.0015 (6)	−0.0045 (6)
C35	0.0151 (8)	0.0190 (8)	0.0137 (7)	0.0006 (6)	0.0006 (6)	−0.0049 (6)
C36	0.0178 (8)	0.0227 (8)	0.0175 (8)	−0.0021 (6)	−0.0017 (6)	−0.0051 (6)
C37	0.0179 (8)	0.0295 (9)	0.0180 (8)	0.0042 (7)	−0.0050 (7)	−0.0066 (7)
C38	0.0268 (9)	0.0219 (8)	0.0208 (8)	0.0086 (7)	−0.0059 (7)	−0.0034 (7)
C39	0.0239 (9)	0.0179 (8)	0.0206 (8)	0.0016 (6)	−0.0035 (7)	−0.0042 (6)
C40	0.0176 (8)	0.0182 (7)	0.0143 (7)	0.0010 (6)	−0.0007 (6)	−0.0035 (6)
C41	0.0184 (8)	0.0154 (7)	0.0175 (8)	0.0002 (6)	−0.0007 (6)	−0.0060 (6)
C42	0.0157 (8)	0.0145 (7)	0.0173 (7)	−0.0006 (6)	−0.0015 (6)	−0.0037 (6)
C43	0.0143 (8)	0.0156 (7)	0.0181 (8)	−0.0023 (6)	−0.0001 (6)	−0.0044 (6)
C44	0.0142 (8)	0.0172 (7)	0.0152 (7)	−0.0018 (6)	−0.0016 (6)	−0.0031 (6)
C45	0.0163 (8)	0.0209 (8)	0.0178 (8)	−0.0020 (6)	−0.0030 (6)	−0.0028 (6)
C46	0.0135 (8)	0.0205 (8)	0.0172 (8)	0.0000 (6)	−0.0015 (6)	−0.0031 (6)
C47	0.0274 (9)	0.0273 (9)	0.0250 (9)	0.0000 (7)	−0.0126 (7)	−0.0046 (7)
C48	0.0392 (11)	0.0306 (9)	0.0283 (9)	−0.0039 (8)	−0.0181 (8)	−0.0067 (8)
C49	0.0205 (8)	0.0216 (8)	0.0203 (8)	0.0031 (6)	−0.0056 (7)	−0.0028 (6)
C50	0.0210 (9)	0.0182 (8)	0.0290 (9)	0.0023 (6)	−0.0063 (7)	0.0002 (7)
C51	0.0345 (10)	0.0175 (8)	0.0195 (8)	0.0003 (7)	−0.0092 (7)	−0.0057 (6)
C52	0.0345 (10)	0.0226 (8)	0.0238 (9)	0.0005 (7)	−0.0068 (8)	−0.0112 (7)
C53	0.0325 (10)	0.0204 (8)	0.0351 (10)	0.0044 (7)	−0.0099 (8)	−0.0146 (7)
C54	0.0298 (9)	0.0133 (8)	0.0325 (9)	0.0022 (7)	−0.0081 (8)	−0.0052 (7)
C55	0.0210 (8)	0.0139 (7)	0.0227 (8)	−0.0022 (6)	−0.0059 (7)	−0.0012 (6)
C56	0.0185 (8)	0.0171 (8)	0.0272 (9)	0.0004 (6)	−0.0061 (7)	−0.0095 (7)
C57	0.0219 (9)	0.0235 (8)	0.0289 (9)	−0.0003 (7)	−0.0011 (7)	−0.0129 (7)
C58	0.0294 (9)	0.0245 (8)	0.0271 (9)	−0.0039 (7)	−0.0041 (7)	−0.0134 (7)
C59	0.0254 (9)	0.0246 (8)	0.0276 (9)	−0.0052 (7)	−0.0073 (7)	−0.0100 (7)
C60	0.0168 (8)	0.0169 (7)	0.0237 (8)	−0.0040 (6)	−0.0039 (7)	−0.0044 (6)

### 2,3-Diethyl-5,12-bis(piperidin-1-yl)naphtho[2,3-g]quinoxaline-6,11-dione (IV) Geometric parameters (Å, º)

**Table:**

O1—C4	1.233 (2)	C25—H25B	0.9900
O2—C11	1.2316 (18)	C25—H25A	0.9900
N1—C1	1.362 (2)	C26—H26A	0.9900
N1—C16	1.315 (2)	C26—H26B	0.9900
N2—C14	1.3674 (19)	C27—H27A	0.9900
N2—C15	1.315 (2)	C27—H27B	0.9900
N3—C2	1.3809 (19)	C28—H28A	0.9900
N3—C21	1.457 (2)	C28—H28B	0.9900
N3—C25	1.466 (2)	C29—H29A	0.9900
N4—C13	1.3870 (19)	C29—H29B	0.9900
N4—C26	1.4575 (19)	C30—H30B	0.9900
N4—C30	1.466 (2)	C30—H30A	0.9900
C1—C2	1.448 (2)	C31—C32	1.450 (2)
C1—C14	1.406 (2)	C31—C44	1.407 (2)
C2—C3	1.390 (2)	C32—C33	1.402 (2)
C3—C4	1.477 (2)	C33—C34	1.470 (2)
C3—C12	1.455 (2)	C33—C42	1.449 (2)
O3—C34	1.2318 (18)	C34—C35	1.493 (2)
C4—C5	1.483 (2)	C35—C36	1.389 (2)
O4—C41	1.2286 (18)	C35—C40	1.398 (2)
C5—C6	1.399 (2)	C36—C37	1.386 (2)
C5—C10	1.400 (2)	C37—C38	1.391 (2)
C6—C7	1.382 (2)	C38—C39	1.380 (2)
C7—C8	1.391 (2)	C39—C40	1.397 (2)
C8—C9	1.380 (2)	C40—C41	1.485 (2)
C9—C10	1.393 (2)	C41—C42	1.478 (2)
C10—C11	1.492 (2)	C42—C43	1.391 (2)
C11—C12	1.473 (2)	C43—C44	1.449 (2)
C12—C13	1.394 (2)	C45—C46	1.432 (2)
C13—C14	1.447 (2)	C45—C47	1.507 (2)
C15—C16	1.425 (2)	C46—C49	1.511 (2)
C15—C17	1.509 (2)	C47—C48	1.509 (2)
C16—C19	1.504 (2)	C49—C50	1.519 (2)
C17—C18	1.516 (2)	C51—C52	1.526 (2)
C19—C20	1.514 (2)	C52—C53	1.520 (2)
C21—C22	1.519 (2)	C53—C54	1.524 (2)
C22—C23	1.518 (3)	C54—C55	1.518 (2)
C23—C24	1.526 (3)	C56—C57	1.524 (2)
C24—C25	1.518 (2)	C57—C58	1.522 (2)
C26—C27	1.525 (2)	C58—C59	1.527 (2)
C27—C28	1.524 (2)	C59—C60	1.515 (2)
C28—C29	1.520 (2)	C36—H36	0.9500
C29—C30	1.520 (2)	C37—H37	0.9500
N5—C31	1.3663 (19)	C38—H38	0.9500
N5—C46	1.316 (2)	C39—H39	0.9500
N6—C45	1.317 (2)	C47—H47A	0.9900
C6—H6	0.9500	C47—H47B	0.9900
N6—C44	1.3631 (19)	C48—H48A	0.9800
N7—C32	1.3822 (19)	C48—H48B	0.9800
C7—H7	0.9500	C48—H48C	0.9800
N7—C51	1.4565 (19)	C49—H49A	0.9900
N7—C55	1.4661 (19)	C49—H49B	0.9900
C8—H8	0.9500	C50—H50A	0.9800
N8—C43	1.3792 (19)	C50—H50B	0.9800
N8—C56	1.457 (2)	C50—H50C	0.9800
N8—C60	1.464 (2)	C51—H51A	0.9900
C9—H9	0.9500	C51—H51B	0.9900
C17—H17B	0.9900	C52—H52A	0.9900
C17—H17A	0.9900	C52—H52B	0.9900
C18—H18A	0.9800	C53—H53A	0.9900
C18—H18B	0.9800	C53—H53B	0.9900
C18—H18C	0.9800	C54—H54A	0.9900
C19—H19A	0.9900	C54—H54B	0.9900
C19—H19B	0.9900	C55—H55A	0.9900
C20—H20C	0.9800	C55—H55B	0.9900
C20—H20A	0.9800	C56—H56A	0.9900
C20—H20B	0.9800	C56—H56B	0.9900
C21—H21A	0.9900	C57—H57A	0.9900
C21—H21B	0.9900	C57—H57B	0.9900
C22—H22B	0.9900	C58—H58A	0.9900
C22—H22A	0.9900	C58—H58B	0.9900
C23—H23A	0.9900	C59—H59A	0.9900
C23—H23B	0.9900	C59—H59B	0.9900
C24—H24A	0.9900	C60—H60A	0.9900
C24—H24B	0.9900	C60—H60B	0.9900
			
C1—N1—C16	118.72 (13)	C30—C29—H29B	109.00
C14—N2—C15	118.47 (13)	H29A—C29—H29B	108.00
C2—N3—C21	121.17 (13)	C30—C29—H29A	109.00
C2—N3—C25	125.19 (13)	N4—C30—H30A	110.00
C21—N3—C25	112.55 (12)	N4—C30—H30B	110.00
C13—N4—C26	121.95 (12)	C29—C30—H30B	110.00
C13—N4—C30	122.39 (12)	H30A—C30—H30B	108.00
C26—N4—C30	112.94 (12)	C29—C30—H30A	110.00
N1—C1—C2	118.83 (14)	N5—C31—C32	118.37 (13)
N1—C1—C14	120.44 (14)	N5—C31—C44	120.43 (13)
C2—C1—C14	120.73 (13)	C32—C31—C44	121.15 (13)
N3—C2—C1	120.25 (13)	N7—C32—C31	119.88 (13)
N3—C2—C3	122.22 (13)	N7—C32—C33	122.91 (13)
C1—C2—C3	117.03 (13)	C31—C32—C33	116.99 (13)
C2—C3—C4	121.26 (13)	C32—C33—C34	121.38 (13)
C2—C3—C12	121.05 (13)	C32—C33—C42	120.31 (14)
C4—C3—C12	117.53 (13)	C34—C33—C42	117.58 (13)
O1—C4—C3	122.55 (15)	O3—C34—C33	123.23 (13)
O1—C4—C5	119.81 (14)	O3—C34—C35	118.34 (14)
C3—C4—C5	117.62 (14)	C33—C34—C35	118.33 (13)
C4—C5—C6	119.94 (14)	C34—C35—C36	119.34 (13)
C4—C5—C10	120.64 (14)	C34—C35—C40	120.87 (14)
C6—C5—C10	119.40 (15)	C36—C35—C40	119.75 (14)
C5—C6—C7	120.19 (15)	C35—C36—C37	120.24 (14)
C6—C7—C8	120.12 (15)	C36—C37—C38	120.00 (15)
C7—C8—C9	120.22 (16)	C37—C38—C39	120.27 (15)
C8—C9—C10	120.23 (15)	C38—C39—C40	120.06 (15)
C5—C10—C9	119.81 (14)	C35—C40—C39	119.69 (14)
C5—C10—C11	120.50 (14)	C35—C40—C41	120.34 (13)
C9—C10—C11	119.59 (14)	C39—C40—C41	119.94 (13)
O2—C11—C10	118.59 (14)	O4—C41—C40	119.99 (13)
O2—C11—C12	123.02 (13)	O4—C41—C42	122.83 (14)
C10—C11—C12	118.33 (13)	C40—C41—C42	117.14 (13)
C3—C12—C11	117.54 (13)	C33—C42—C41	117.83 (13)
C3—C12—C13	120.24 (14)	C33—C42—C43	121.14 (13)
C11—C12—C13	121.43 (13)	C41—C42—C43	120.91 (13)
N4—C13—C12	123.60 (14)	N8—C43—C42	122.42 (13)
N4—C13—C14	118.99 (13)	N8—C43—C44	119.96 (13)
C12—C13—C14	117.22 (13)	C42—C43—C44	117.09 (13)
N2—C14—C1	120.16 (14)	N6—C44—C31	120.42 (13)
N2—C14—C13	118.38 (13)	N6—C44—C43	118.61 (13)
C1—C14—C13	121.41 (13)	C31—C44—C43	120.97 (13)
N2—C15—C16	121.24 (14)	N6—C45—C46	121.02 (14)
N2—C15—C17	118.70 (14)	N6—C45—C47	118.62 (14)
C16—C15—C17	120.03 (13)	C46—C45—C47	120.35 (14)
N1—C16—C15	120.91 (14)	N5—C46—C45	121.07 (14)
N1—C16—C19	118.23 (14)	N5—C46—C49	118.68 (14)
C15—C16—C19	120.85 (14)	C45—C46—C49	120.22 (13)
C15—C17—C18	114.69 (14)	C45—C47—C48	114.68 (14)
C16—C19—C20	114.36 (14)	C46—C49—C50	113.89 (14)
N3—C21—C22	110.29 (13)	N7—C51—C52	110.35 (13)
C21—C22—C23	112.30 (14)	C51—C52—C53	110.71 (13)
C22—C23—C24	109.93 (14)	C52—C53—C54	109.47 (13)
C23—C24—C25	110.26 (15)	C53—C54—C55	111.00 (13)
N3—C25—C24	110.77 (14)	N7—C55—C54	110.53 (12)
N4—C26—C27	109.74 (13)	N8—C56—C57	109.61 (13)
C26—C27—C28	111.05 (13)	C56—C57—C58	111.50 (13)
C27—C28—C29	109.84 (13)	C57—C58—C59	110.26 (14)
C28—C29—C30	110.81 (14)	C58—C59—C60	110.96 (13)
N4—C30—C29	110.34 (13)	N8—C60—C59	110.74 (13)
C31—N5—C46	118.44 (13)	C35—C36—H36	120.00
C5—C6—H6	120.00	C37—C36—H36	120.00
C7—C6—H6	120.00	C36—C37—H37	120.00
C44—N6—C45	118.56 (13)	C38—C37—H37	120.00
C32—N7—C51	121.25 (12)	C37—C38—H38	120.00
C32—N7—C55	123.08 (12)	C39—C38—H38	120.00
C51—N7—C55	112.71 (12)	C38—C39—H39	120.00
C6—C7—H7	120.00	C40—C39—H39	120.00
C8—C7—H7	120.00	C45—C47—H47A	109.00
C56—N8—C60	112.97 (12)	C45—C47—H47B	109.00
C43—N8—C56	121.01 (13)	C48—C47—H47A	109.00
C43—N8—C60	123.82 (13)	C48—C47—H47B	109.00
C7—C8—H8	120.00	H47A—C47—H47B	108.00
C9—C8—H8	120.00	C47—C48—H48A	109.00
C8—C9—H9	120.00	C47—C48—H48B	109.00
C10—C9—H9	120.00	C47—C48—H48C	109.00
C15—C17—H17A	109.00	H48A—C48—H48B	109.00
C18—C17—H17A	109.00	H48A—C48—H48C	109.00
C18—C17—H17B	109.00	H48B—C48—H48C	109.00
H17A—C17—H17B	108.00	C46—C49—H49A	109.00
C15—C17—H17B	109.00	C46—C49—H49B	109.00
C17—C18—H18A	109.00	C50—C49—H49A	109.00
C17—C18—H18C	109.00	C50—C49—H49B	109.00
H18A—C18—H18B	109.00	H49A—C49—H49B	108.00
C17—C18—H18B	109.00	C49—C50—H50A	109.00
H18B—C18—H18C	109.00	C49—C50—H50B	109.00
H18A—C18—H18C	109.00	C49—C50—H50C	109.00
C16—C19—H19A	109.00	H50A—C50—H50B	109.00
H19A—C19—H19B	108.00	H50A—C50—H50C	109.00
C20—C19—H19B	109.00	H50B—C50—H50C	109.00
C16—C19—H19B	109.00	N7—C51—H51A	110.00
C20—C19—H19A	109.00	N7—C51—H51B	110.00
C19—C20—H20C	109.00	C52—C51—H51A	110.00
C19—C20—H20B	109.00	C52—C51—H51B	110.00
H20A—C20—H20B	109.00	H51A—C51—H51B	108.00
H20A—C20—H20C	109.00	C51—C52—H52A	110.00
H20B—C20—H20C	109.00	C51—C52—H52B	110.00
C19—C20—H20A	109.00	C53—C52—H52A	110.00
N3—C21—H21A	110.00	C53—C52—H52B	109.00
C22—C21—H21A	110.00	H52A—C52—H52B	108.00
C22—C21—H21B	110.00	C52—C53—H53A	110.00
N3—C21—H21B	110.00	C52—C53—H53B	110.00
H21A—C21—H21B	108.00	C54—C53—H53A	110.00
C21—C22—H22A	109.00	C54—C53—H53B	110.00
C21—C22—H22B	109.00	H53A—C53—H53B	108.00
C23—C22—H22A	109.00	C53—C54—H54A	109.00
C23—C22—H22B	109.00	C53—C54—H54B	109.00
H22A—C22—H22B	108.00	C55—C54—H54A	109.00
C22—C23—H23A	110.00	C55—C54—H54B	109.00
C24—C23—H23A	110.00	H54A—C54—H54B	108.00
C24—C23—H23B	110.00	N7—C55—H55A	110.00
H23A—C23—H23B	108.00	N7—C55—H55B	110.00
C22—C23—H23B	110.00	C54—C55—H55A	110.00
C23—C24—H24A	110.00	C54—C55—H55B	110.00
C25—C24—H24A	110.00	H55A—C55—H55B	108.00
C25—C24—H24B	110.00	N8—C56—H56A	110.00
C23—C24—H24B	110.00	N8—C56—H56B	110.00
H24A—C24—H24B	108.00	C57—C56—H56A	110.00
N3—C25—H25B	109.00	C57—C56—H56B	110.00
N3—C25—H25A	109.00	H56A—C56—H56B	108.00
H25A—C25—H25B	108.00	C56—C57—H57A	109.00
C24—C25—H25A	109.00	C56—C57—H57B	109.00
C24—C25—H25B	109.00	C58—C57—H57A	109.00
C27—C26—H26A	110.00	C58—C57—H57B	109.00
N4—C26—H26A	110.00	H57A—C57—H57B	108.00
C27—C26—H26B	110.00	C57—C58—H58A	110.00
H26A—C26—H26B	108.00	C57—C58—H58B	110.00
N4—C26—H26B	110.00	C59—C58—H58A	110.00
C26—C27—H27A	109.00	C59—C58—H58B	110.00
C26—C27—H27B	109.00	H58A—C58—H58B	108.00
C28—C27—H27B	109.00	C58—C59—H59A	109.00
H27A—C27—H27B	108.00	C58—C59—H59B	109.00
C28—C27—H27A	109.00	C60—C59—H59A	109.00
C27—C28—H28A	110.00	C60—C59—H59B	109.00
C27—C28—H28B	110.00	H59A—C59—H59B	108.00
C29—C28—H28A	110.00	N8—C60—H60A	109.00
C29—C28—H28B	110.00	N8—C60—H60B	109.00
H28A—C28—H28B	108.00	C59—C60—H60A	109.00
C28—C29—H29A	109.00	C59—C60—H60B	110.00
C28—C29—H29B	109.00	H60A—C60—H60B	108.00
			
C16—N1—C1—C2	179.25 (14)	C46—N5—C31—C32	−174.61 (14)
C16—N1—C1—C14	−1.0 (2)	C46—N5—C31—C44	2.8 (2)
C1—N1—C16—C15	1.0 (2)	C31—N5—C46—C45	−2.9 (2)
C1—N1—C16—C19	−177.77 (14)	C31—N5—C46—C49	175.11 (14)
C15—N2—C14—C1	−2.8 (2)	C45—N6—C44—C31	0.6 (2)
C15—N2—C14—C13	174.61 (14)	C45—N6—C44—C43	179.97 (14)
C14—N2—C15—C16	2.7 (2)	C44—N6—C45—C46	−0.6 (2)
C14—N2—C15—C17	−175.36 (14)	C44—N6—C45—C47	178.61 (14)
C21—N3—C2—C1	144.09 (15)	C51—N7—C32—C31	−144.83 (15)
C21—N3—C2—C3	−27.5 (2)	C51—N7—C32—C33	29.6 (2)
C25—N3—C2—C1	−48.7 (2)	C55—N7—C32—C31	56.1 (2)
C25—N3—C2—C3	139.68 (16)	C55—N7—C32—C33	−129.45 (16)
C2—N3—C21—C22	−133.83 (14)	C32—N7—C51—C52	140.55 (14)
C25—N3—C21—C22	57.50 (17)	C55—N7—C51—C52	−58.37 (17)
C2—N3—C25—C24	132.31 (15)	C32—N7—C55—C54	−141.30 (15)
C21—N3—C25—C24	−59.57 (17)	C51—N7—C55—C54	58.02 (17)
C26—N4—C13—C12	−31.0 (2)	C56—N8—C43—C42	27.8 (2)
C26—N4—C13—C14	143.79 (14)	C56—N8—C43—C44	−143.56 (14)
C30—N4—C13—C12	129.01 (16)	C60—N8—C43—C42	−134.16 (16)
C30—N4—C13—C14	−56.2 (2)	C60—N8—C43—C44	54.5 (2)
C13—N4—C26—C27	−139.42 (14)	C43—N8—C56—C57	137.09 (14)
C30—N4—C26—C27	58.87 (17)	C60—N8—C56—C57	−59.11 (16)
C13—N4—C30—C29	139.43 (15)	C43—N8—C60—C59	−137.48 (14)
C26—N4—C30—C29	−58.95 (17)	C56—N8—C60—C59	59.25 (16)
N1—C1—C2—N3	10.8 (2)	N5—C31—C32—N7	−10.2 (2)
N1—C1—C2—C3	−177.20 (14)	N5—C31—C32—C33	174.99 (14)
C14—C1—C2—N3	−168.93 (14)	C44—C31—C32—N7	172.40 (14)
C14—C1—C2—C3	3.1 (2)	C44—C31—C32—C33	−2.4 (2)
N1—C1—C14—N2	1.9 (2)	N5—C31—C44—N6	−1.6 (2)
N1—C1—C14—C13	−175.35 (14)	N5—C31—C44—C43	178.96 (14)
C2—C1—C14—N2	−178.34 (14)	C32—C31—C44—N6	175.67 (14)
C2—C1—C14—C13	4.4 (2)	C32—C31—C44—C43	−3.7 (2)
N3—C2—C3—C4	−27.3 (2)	N7—C32—C33—C34	29.0 (2)
N3—C2—C3—C12	157.34 (14)	N7—C32—C33—C42	−161.12 (14)
C1—C2—C3—C4	160.91 (14)	C31—C32—C33—C34	−156.43 (14)
C1—C2—C3—C12	−14.5 (2)	C31—C32—C33—C42	13.5 (2)
C2—C3—C4—O1	−24.8 (2)	C32—C33—C34—O3	11.5 (2)
C2—C3—C4—C5	157.11 (14)	C32—C33—C34—C35	−172.26 (13)
C12—C3—C4—O1	150.71 (15)	C42—C33—C34—O3	−158.64 (14)
C12—C3—C4—C5	−27.3 (2)	C42—C33—C34—C35	17.6 (2)
C2—C3—C12—C11	−150.87 (14)	C32—C33—C42—C41	156.56 (14)
C2—C3—C12—C13	19.1 (2)	C32—C33—C42—C43	−19.4 (2)
C4—C3—C12—C11	33.6 (2)	C34—C33—C42—C41	−33.1 (2)
C4—C3—C12—C13	−156.44 (14)	C34—C33—C42—C43	150.87 (14)
O1—C4—C5—C6	10.3 (2)	O3—C34—C35—C36	−4.6 (2)
O1—C4—C5—C10	−171.27 (14)	O3—C34—C35—C40	177.78 (14)
C3—C4—C5—C6	−171.59 (14)	C33—C34—C35—C36	178.99 (13)
C3—C4—C5—C10	6.8 (2)	C33—C34—C35—C40	1.4 (2)
C4—C5—C6—C7	177.76 (14)	C34—C35—C36—C37	−177.50 (14)
C10—C5—C6—C7	−0.7 (2)	C40—C35—C36—C37	0.1 (2)
C4—C5—C10—C9	−176.54 (14)	C34—C35—C40—C39	177.48 (14)
C4—C5—C10—C11	7.3 (2)	C34—C35—C40—C41	−4.7 (2)
C6—C5—C10—C9	1.9 (2)	C36—C35—C40—C39	−0.1 (2)
C6—C5—C10—C11	−174.32 (14)	C36—C35—C40—C41	177.76 (14)
C5—C6—C7—C8	−0.9 (2)	C35—C36—C37—C38	0.0 (2)
C6—C7—C8—C9	1.2 (2)	C36—C37—C38—C39	−0.2 (2)
C7—C8—C9—C10	0.0 (2)	C37—C38—C39—C40	0.3 (2)
C8—C9—C10—C5	−1.6 (2)	C38—C39—C40—C35	−0.1 (2)
C8—C9—C10—C11	174.68 (14)	C38—C39—C40—C41	−177.95 (14)
C5—C10—C11—O2	−178.18 (14)	C35—C40—C41—O4	167.23 (14)
C5—C10—C11—C12	−1.0 (2)	C35—C40—C41—C42	−10.7 (2)
C9—C10—C11—O2	5.6 (2)	C39—C40—C41—O4	−14.9 (2)
C9—C10—C11—C12	−177.24 (13)	C39—C40—C41—C42	167.18 (14)
O2—C11—C12—C3	157.57 (14)	O4—C41—C42—C33	−148.11 (15)
O2—C11—C12—C13	−12.3 (2)	O4—C41—C42—C43	27.9 (2)
C10—C11—C12—C3	−19.5 (2)	C40—C41—C42—C33	29.7 (2)
C10—C11—C12—C13	170.68 (14)	C40—C41—C42—C43	−154.27 (14)
C3—C12—C13—N4	163.81 (14)	C33—C42—C43—N8	−158.85 (14)
C3—C12—C13—C14	−11.0 (2)	C33—C42—C43—C44	12.8 (2)
C11—C12—C13—N4	−26.6 (2)	C41—C42—C43—N8	25.3 (2)
C11—C12—C13—C14	158.56 (14)	C41—C42—C43—C44	−163.10 (14)
N4—C13—C14—N2	7.3 (2)	N8—C43—C44—N6	−9.1 (2)
N4—C13—C14—C1	−175.33 (14)	N8—C43—C44—C31	170.33 (14)
C12—C13—C14—N2	−177.57 (14)	C42—C43—C44—N6	179.09 (14)
C12—C13—C14—C1	−0.2 (2)	C42—C43—C44—C31	−1.5 (2)
N2—C15—C16—N1	−1.9 (2)	N6—C45—C46—N5	1.9 (2)
N2—C15—C16—C19	176.83 (14)	N6—C45—C46—C49	−176.09 (14)
C17—C15—C16—N1	176.18 (14)	C47—C45—C46—N5	−177.38 (14)
C17—C15—C16—C19	−5.1 (2)	C47—C45—C46—C49	4.7 (2)
N2—C15—C17—C18	14.6 (2)	N6—C45—C47—C48	−1.9 (2)
C16—C15—C17—C18	−163.53 (14)	C46—C45—C47—C48	177.32 (14)
N1—C16—C19—C20	4.9 (2)	N5—C46—C49—C50	−11.8 (2)
C15—C16—C19—C20	−173.81 (14)	C45—C46—C49—C50	166.16 (14)
N3—C21—C22—C23	−54.60 (17)	N7—C51—C52—C53	56.87 (18)
C21—C22—C23—C24	53.28 (18)	C51—C52—C53—C54	−55.34 (18)
C22—C23—C24—C25	−53.88 (18)	C52—C53—C54—C55	55.07 (18)
C23—C24—C25—N3	56.99 (17)	C53—C54—C55—N7	−55.94 (18)
N4—C26—C27—C28	−56.46 (18)	N8—C56—C57—C58	56.07 (17)
C26—C27—C28—C29	54.93 (19)	C56—C57—C58—C59	−53.61 (18)
C27—C28—C29—C30	−54.54 (19)	C57—C58—C59—C60	52.82 (18)
C28—C29—C30—N4	56.00 (18)	C58—C59—C60—N8	−55.07 (17)

### 2,3-Diethyl-5,12-bis(piperidin-1-yl)naphtho[2,3-g]quinoxaline-6,11-dione (IV) Hydrogen-bond geometry (Å, º)

**Table:**

*D*—H···*A*	*D*—H	H···*A*	*D*···*A*	*D*—H···*A*
C21—H21*A*···O1	0.99	2.39	2.826 (2)	106
C25—H25*A*···N1	0.99	2.28	2.872 (2)	117
C26—H26*A*···O2	0.99	2.23	2.7484 (19)	111
C30—H30*A*···N2	0.99	2.32	2.888 (2)	115
C51—H51*B*···O3	0.99	2.22	2.752 (2)	113
C55—H55*B*···N5	0.99	2.31	2.910 (2)	118
C56—H56*A*···O3^i^	0.99	2.54	3.1765 (19)	122
C56—H56*B*···O4	0.99	2.34	2.822 (2)	109
C60—H60*B*···N6	0.99	2.34	2.910 (2)	116

Symmetry code: (i) −*x*, −*y*+1, −*z*+1.
